# Effects of Methyl Jasmonate on Acute Stress Responses in Mice Subjected to Forced Swim and Anoxic Tests

**DOI:** 10.3797/scipharm.1504-04

**Published:** 2015-06-11

**Authors:** Oritoke M. Aluko, Solomon Umukoro, Olajide S. Annafi, Folashade A. Adewole, Osarume Omorogbe

**Affiliations:** Department of Pharmacology and Therapeutics, College of Medicine, University of Ibadan, Ibadan, Nigeria

**Keywords:** Methyl jasmonate, Forced swim endurance test, Anoxic tolerance test, Adaptogen

## Abstract

Methyl jasmonate (MJ) is an anti-stress hormone released by plants in response to external stressors and aids adaptation to stress. In this study, we evaluated the anti-stress activity of MJ using the forced swim endurance test (FSET) and anoxic tolerance test in mice. Male Swiss mice were given MJ (25–100 mg/kg, i.p) 30 min before the FSET and anoxic test were carried out. The first occurrence of immobility, duration of immobility, time spent in active swimming, and latency to exhaustion were assessed in the FSET. The onset to anoxic convulsion was measured in the anoxic tolerance test. MJ significantly (p < 0.05) delayed the first occurrence of immobility and shortened the period of immobility, which indicates anti-stress property. MJ also increased the time spent in active swimming and prolonged the latency to exhaustion, which further suggests anti-stress activity. In addition, it also exhibited anti-stress property as evidenced by prolonged latency to first appearance of anoxic convulsions. The results of this study suggest that MJ demonstrated anti-stress activity and may be useful as an energizer in times of body weakness or exhaustion. Although more studies are necessary before concluding on how MJ exerts its anti-stress activity, the present data suggest an action similar to adaptogens in boosting energy and resilience in the face of stress.

## Introduction

Stress is an integral part of human life and is considered to be any condition which results in perturbation of the body’s homeostasis [1–4]. During extreme stress situations, the homeostatic mechanisms of the organism become deficient and the survival of the organism is thereby threatened [3–4]. Response to stress includes adaptation, but when sustained over a long period, it leads to ill health and increased susceptibility to illnesses [4]. Indeed, stress has been postulated to be involved in the pathogenesis of a variety of diseases including arthritis, hypertension, peptic ulcer, diabetes, reproductive dysfunctions, cancer, immune dysfunctions, depression, and neurodegenerative disorders [4–6]. Although stress has been known to have deleterious effects on the body for over a century, drugs which could ameliorate its pathological impacts are yet to be discovered. The conventional use of stimulants and anabolic steroids for stress management are ineffective in combating the multiple effects of stressors and are also associated with serious adverse effects [5–7]. However, in contrast to these conventional agents, adaptogens, which are compounds of plant origin, are devoid of such adverse effects [6–9].

Adaptogens possess multipronged mechanisms of action and act nonspecifically to provide resistance against stressful conditions by regulating the various elements involved in the stress response system [6–11]. Adaptogens have been described as “medicines for healthy people” and are believed to boost energy and resilience in the face of stress; they promote physical and mental performance, improve defense mechanisms of the body, and enhance longevity [11]. A number of medicinal plants such as *Rhodiola rose, Panax ginseng, Ginkgo biloba, Ocimum sanctum, Withania somnifera, Pausinystalia yohimbe, and the* Phyllanthus species have been shown to possess adaptogenic properties and some of them are available commercially for stress management [7–12].

Methyl jasmonate (MJ) belongs to the jasmonate family and it helps to provide resistance for plants against external stressors [13]. MJ was first isolated from the essential oil of *Jasminum grandiflorum*, a medicinal plant found in most tropical regions of the world, which is now available commercially through chemical synthesis [14]. MJ is a cyclopentanoic compound with high lipid solubility, a property that encourages its bioavailability in the brain [15]. Previous studies have reported that MJ possess anticancer, antinociceptive, anti-aggressive, and antidepressant activities [13–18]. Considering the fact that MJ provides a defensive mechanism for plants against stress suggests that it might also exhibit an adaptogenic property in providing resistance against stressful conditions in humans. This preliminary investigation was carried out to evaluate the possible anti-stress properties of methyl jasmonate in mice.

## Experimental

### Experimental Animals

Male Swiss mice (20–25 g) obtained from the Central Animal House, University of Ibadan were used in the study. They were housed in plastic cages at room temperature and were fed with rodent pellet diet and water *ad libitum*. All experimental animals were handled in accordance with the US National Institutes of Health (NIH) Guideline for the Care and Use of Laboratory Animals.

### Drugs and Treatment

Methyl jasmonate (Sigma-Aldrich, St. Louis, USA) and yohimbine (Sigma, USA) were used in the study. MJ was dissolved in ethanol and this solution was further diluted with distilled water. The final concentration of ethanol in the solution used for the study did not exceed 1%. Yohimbine was dissolved in distilled water immediately before use. The doses of MJ used in the study were selected based on the results obtained from previous investigations [16-18]. The animals were divided into five groups (n = 6) and pretreated intraperitonially (i.p.) with MJ (25, 50, and 100 mg/kg), yohimbine (5 mg/kg), or a vehicle (10 mL/kg of 1.0% ethanol) 30 min prior to the commencement of the experiment.

### Forced Swim Endurance Test (FSET)

This was evaluated according to the method previously described by Porsolt *et al*. [19]. Each animal was forced to swim individually for 15 min in a glass jar of height 20 cm, diameter 10 cm, and filled with fresh water to a depth of 15 cm and the water was maintained at room temperature. The parameters measured were first occurrence of immobility (the period the animal swims continuously before the first pause of swimming activity), duration of immobility (the total time during which the animal is immobile), total time spent in active swimming (the total duration during which the animal swims throughout the experimental period), and latency to exhaustion (the period when the animal starts to sink).

### Anoxic Tolerance Test

This test was carried out based on the method previously described by Caillard *et al*. [20]. The animals were individually placed in an air-tight hermetic vessel of 250 mL capacity. Thereafter, the anoxic tolerance time was recorded and the animals were immediately removed from the vessel for recovery. The anoxic tolerance time was defined as the latency to the first appearance of anoxic convulsions [20].

### Statistical Analysis

The data were analyzed using Graph Pad Prism software version 5.0 and were expressed as the mean ± S.E.M. Statistical analysis of the data was done using one-way ANOVA, followed by the Newman-Keuls post hoc test. Values were considered statistically significant at p < 0.05.

## Results

### Effect of Methyl Jasmonate on the Performance of Mice in Forced Swim Endurance Test

One-way ANOVA revealed that there were significant differences between treatment groups: latency to immobility [F (4, 25) = 30.99, p<0.0001]; total swimming time [F (4, 25) = 39.44, p<0.0001]; duration of immobility [F (4, 25) = 13.21, p< 0.0001]; and latency to exhaustion [F (4, 25) = 32.54, p< 0.0001]. The post hoc analysis by the Newman Keuls test showed that MJ (25, 50, and 100 mg/kg, i.p) significantly prolonged the latency to immobility and increased the time spent in active swimming when compared with the vehicle, suggesting anti-stress property (Table 1). Similar effects were also produced by the reference drug, yohimbine (5 mg/kg, i.p) in comparison with the vehicle (Table 1).

**Tab. 1:**
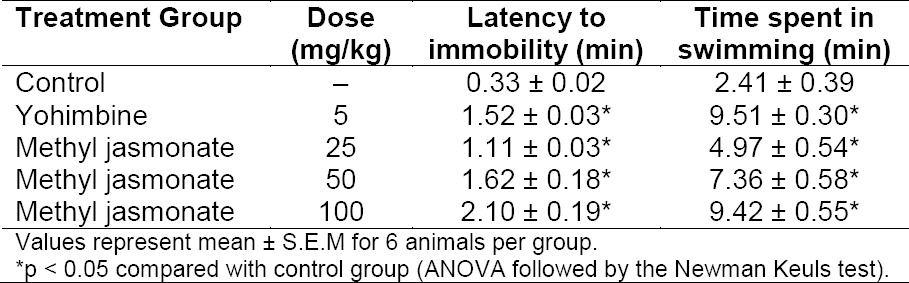
Effect of methyl jasmonate on active and total swimming time in the forced swim test in mice

**Fig. 1:**
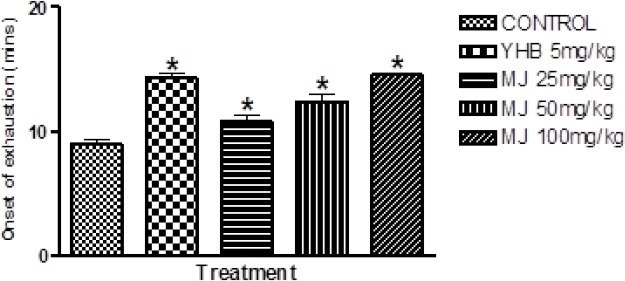
Effect of methyl jasmonate on duration of immobility in the forced swim endurance test in mice. Each column represents the mean ± S.E.M for 6 animals per group. *p < 0.05 compared with the control (ANOVA followed by the Newman Keuls test)

**Fig. 2:**
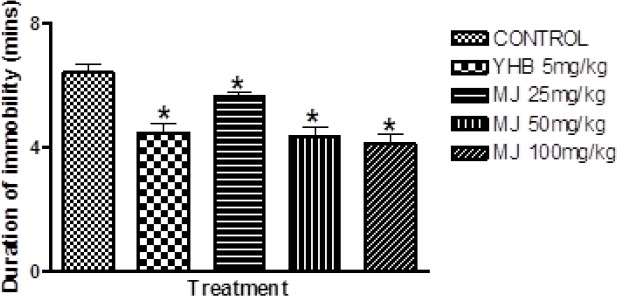
Effect of methyl jasmonate on the latency to exhaustion in the forced swim endurance test in mice. Each column represents the mean ± S.E.M for 6 animals per group. *p < 0.05 compared with the control (ANOVA followed by the Newman Keuls test)

As shown in Figures 1 and 2, MJ (25, 50, and 100 mg/kg) significantly (p < 0.05) decreased the duration of immobility and delayed the latency to exhaustion when compared with the vehicle, which further suggest anti-stress activity. In a similar manner, yohimbine (5 mg/kg, i.p), also shortened the period of immobility and prolonged the latency to exhaustion in comparison with the vehicle (Figures 1 & 2).

### Effect of Methyl Jasmonate on the Performance of Mice in the Anoxic Tolerance Test

**Fig. 3:**
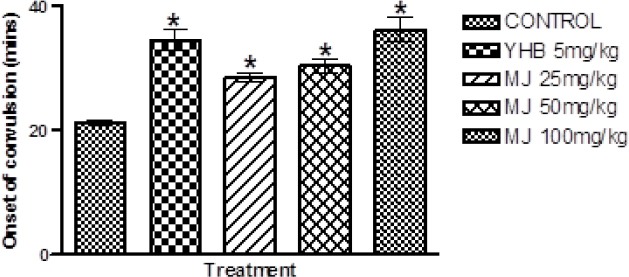
Effect of methyl jasmonate on the latency to convulsion in the anoxic tolerance test in mice. Each column represents the mean ± S.E.M for 6 animals per group. *p < 0.05 compared with the vehicle (ANOVA followed by the Newman Keuls test)

The effect of MJ on the latency to anoxic convulsion in the anoxic tolerance test in mice is shown in Figure 3. One-way ANOVA revealed that there were significant differences between treatment groups: latency to anoxic convulsions [F (4, 25) = 20.29, p < 0.0001]. Post hoc analysis by the Newman Keuls test revealed that MJ (25, 50, and 100 mg/kg, i.p) significantly delayed the latency to anoxic convulsion in comparison with the vehicle, which indicates anti-stress effect (Fig. 3). Similarly, yohimbine also significantly prolonged the latency to anoxic convulsions in comparison with the vehicle (Fig. 3).

## Discussion

The results of this study showed that MJ demonstrated adaptogenic-like property during acute stress responses in the forced swim endurance test in mice. The forced swim endurance test is an animal model widely used for evaluation of novel compounds with anti-stress/adaptogenic activity in rodents [21, 22]. The parameters indicative of the acute stress response in the FSET include the first occurrence of immobility, duration of immobility, time spent in active swimming, and latency to exhaustion. The first occurrence of immobility signals the onset of tiredness/fatigue, hopelessness, or despair. Thus, the delay in latency to the first appearance of immobility is an indication of endurance promoting effect or anti-stress property.

It is a well-known phenomenon that exposure of rodents to inescapable aversive stressful situations like the FSET induces a characteristic feature of immobility, which reflects a loss of stamina and state of despair or hopelessness in rodents [2, 21–24]. Thus, the anti-stress property is envisaged when a test compound decreases the duration of immobility in the FSET [23, 24]. Also, a compound may be judged as having an anti-stress property if it increases the duration of time spent in active swimming and delays the latency to exhaustion in the FSET. The phase of exhaustion in the stress response occurs whenever there is a breakdown in the ability of the organisms to adapt to the challenging aversive situation [2, 24]. At this stage, the organism has reached its wilt end, as it can no longer cope and disease pathologies set in as a result of the breakdown in homeostatic mechanisms of the organism [21–24]. The findings that MJ significantly modified the components of the acute stress responses assessed in the FSET, especially in delaying the onset of exhaustion, suggest an action that resembles adaptogens. Generally, adaptogens are known to help the body to cope more effectively with stress and thereby delay the onset of exhaustion [24, 25]. The major warning signs that signal the onset of exhaustion include feelings of hopelessness and reduced energy or stamina, which are the major characteristic features of the stage of immobility in the FSET [21–24]. Thus, the anti-immobility effect demonstrated by MJ in this study further supports the notion that it has adaptogenic-like property in mice.

The anoxic tolerance test was further employed to evaluate the anti-stress/adaptogenic property of MJ based on the prolongation in the latency to anoxic convulsions in mice [20]. The manifestation of convulsive episodes in the anoxic tolerance test is related to acute oxygen deprivation and accumulation of carbon monoxide in the brain [26]. The brain cells are very sensitive to oxygen deprivation and increased concentrations of carbon monoxide, which may lead to neuronal cell death and loss of functions [27]. Compounds with adaptogenic activity are known to delay the latency to anoxic convulsions in mice [23]. Our results showed that MJ exhibited anti-stress activity, as indicated by the increase in duration of anoxic stress tolerance time in mice. The anti-anoxic effect of MJ might be related to increased cerebral resistance and efficient utilization of oxygen during the acute hypoxic stress response in mice. However, this is subject to further experimental investigations and validations.

Although more studies are necessary before concluding on how MJ exerts its anti-stress activity, the present data suggests that it may be acting like yohimbine, in boosting energy and resilience in the face of acute stress. Yohimbine is an alkaloid, obtained from *Pausinystalia yohimbe*, which has been listed among plants with adatogenic properties [28, 29]. Adaptogens are active compounds of plant origin that act nonspecifically to provide resistance against stressful conditions by regulating the various molecular pathways involved in the stress response system [6, 10, 30]. Adaptogens are known to boost the energy needed by the organism to effectively cope with aversive stressful situations. They enable the body’s cells to access more energy and to utilize oxygen more efficiently [8, 9, 30]. The anti-fatigue and endurance-promoting effect of yohimbine is related to the activation of the sympathetic nervous system as a consequence of antagonism of pre-synaptic α_2_-adrenergic receptors [31]. The sympathetic nervous system provides the needed energy to combat the acute stress response, and also for the fight or flight response. In addition, yohimbine has been reported to promote the release of adrenaline and corticosteroids from the adrenal glands, which further strengthen the organism to more effectively cope with acute stress reactions [23, 32]. However, Panossian and Wikman [25] reported that adaptogens do not work only via the hypothalamic posterior adrenal axis and sympathetic adrenal system, but also modulate cellular regulatory elements such as molecular chaperons, stress-activated c-Jun N-terminal protein kinase, and Forkhead Box O transcription factor DAF-16, which are the key mediators of the stress response. Studies have also shown that pretreatment of animals with yohimbine attenuated acute stress responses by promoting the release of galanin [33] and inhibiting the activation of the caspases-9 and Bcl-2 family of apoptotic regulatory proteins [32]. However, more studies are necessary before any meaningful conclusions can be drawn on how MJ exerts its anti-stress property in mice.

## Conclusion

The results of this study suggest that MJ has an adaptogenic-like property and may be useful in mitigating the deleterious effect of stress. Although more studies are necessary before concluding on how MJ exerts its anti-stress activity, the present data suggest it may be acting like adaptogens in boosting energy and resilience in the face of stress.
